# Unique extrication structure in a new megaspilid, *Dendrocerus scutellaris* Trietsch & Mikó (Hymenoptera: Megaspilidae)

**DOI:** 10.3897/BDJ.6.e22676

**Published:** 2018-01-30

**Authors:** Carolyn Trietsch, István Mikó, David G. Notton, Andrew R. Deans

**Affiliations:** 1 Frost Entomological Museum, Penn State University, University Park, PA, United States of America; 2 Natural History Museum, London, United Kingdom

**Keywords:** Ceraphronoidea, morphology, systematics, taxonomy, eclosion

## Abstract

**Background:**

A new species, *Dendrocerus scutellaris* Trietsch & Mikó (Hymenoptera: Megaspilidae), is described here from male and female specimens captured in Costa Rica. This species is the only known ceraphronoid wasp with a straight mandibular surface and raised dorsal projections on the scutellum, called the mesoscutellar comb. It is hypothesised that the function of the mesoscutellar comb is to aid the emergence of the adult from the host, especially since the mandibles lack a pointed surface to tear open the pupal case. The authors also provide phenotypic data in a semantic form to facilitate data integration and accessibility across taxa and provide an updated phenotype bank of morphological characters for megaspilid taxonomic treatments. In updating this phenotype bank, the authors continue to make taxonomic data accessible to future systematic efforts focusing on Ceraphronoidea.

**New information:**

A new species, *Dendrocerus scutellaris* (Hymenoptera: Megaspilidae) Trietsch & Mikó, is described from both male and female specimens captured in Costa Rica.

## Introduction

Ceraphronoidea is a relatively small superfamily of parasitoid wasps with a worldwide distribution (Johnson and Musetti 2004). The superfamily is composed of two extant families: Ceraphronidae and Megaspilidae. *Dendrocerus* Ratzeburg, 1852 is the second most diverse megaspilid genus with approximately 118 species described worldwide. *Dendrocerus* is also the most well-known megaspilid genus due to the agriculturally relevant species *D. carpenteri* (Curtis, 1829), which is used as a model organism to study parasitoid behaviour and ecology (Chow and Mackauer 1999, Mackauer and Chow 2015, Mackauer 2017, Nakashima et al. 2016, Schwörer et al. 1999), but much work remains to be done on the life history and taxonomy of the group.

Eclosion is the adult emergence from the pupal case in holometabolous insects. In most holometabolous insects, the tearing of the pupal case is achieved by the movement of the insect and the increased hemolymph pressure caused by muscle contractions (Gullan and Cranston 2010, Zdárek and Denlinger 1992). Insects that are also protected by a cocoon or puparium may cut or push their way out with their mandibles and legs, or rely on specialized structures such as projections on the head or backward-facing spines on the dorsal surface of the abdomen (Gullan and Cranston 2010). Packard Jr (1878) reported spines called the *sector coconis* present at the base of the forewings in *Bombyx mori* (Linnaeus, 1758) and several species of Saturniidae (Lepidoptera) and observed the spine in use by an emerging *Actias luna* (Linnaeus, 1758). Abdominal spines and frontal protuberances serving as “cocoon-cutters” have also been reported on male Psychidae (Lepidoptera); these structures are absent from females, which do not extricate themselves from the pupal case Davis 1975.

In Hymenoptera, wood-boring families have been observed to have specialised structures for extricating themselves from the pupal chambers inside wood where they develop (Vilhelmsen and Turrisi 2011). Ibaliidae, Orussidae and Stephanidae have upward- or backward-facing cuticular processes situated anterodorsally on the head. Emerging wasps use these structures to anchor their heads while chewing through wood with their mandibles, but they could also be used to clear debris or pull themselves through the wooden galleries (Vilhelmsen and Turrisi 2011). Similar cuticular processes and spines on the mesosomata of Ibaliidae, Stephanidae, Aulacidae, the cynipoid family Liopteridae and the ichneumonid subfamily Rhyssinae may function in the same way (Turrisi and Vilhelmsen 2010, Vilhelmsen and Turrisi 2011, Van Noort and Buffington 2013, Rousse and Van Noort 2014). Along with wood-boring families, mesoscutal spines are found in Platygastroidea (Austin 1984, Johnson et al. 2008); instead of assisting in emergence from wooden galleries, these spines may assist wasps in emerging from the remains of their hosts.

Here, a new species is described of *Dendrocerus* captured in Costa Rica characterised by the presence of a straight mandibular edge and the mesoscutellar comb, which could aid in emergence. These two structures have never before been recorded in Ceraphronoidea and are discussed here for the first time.

## Materials and methods

Point-mounted specimens were borrowed from the Natural History Museum (NHMUK) in London, United Kingdom. Specimen data is provided in Suppl. material 1. All specimen data was also entered into a Microsoft Excel spreadsheet template from GBIF so that the data could be published on GBIF using the Integrated Publishing Toolkit (https://www.gbif.org/news/82852/new-darwin-core-spreadsheet-templates-simplify-data-preparation-and-publishing). Specimens are deposited at the Natural History Museum in London, United Kingdom (NHMUK) and at the Frost Entomological Museum, University Park, PA, USA (PSUC).

Point-mounted and glycerine-dissected specimens were examined using an Olympus SZX16 stereomicroscope with an Olympus SDF PLAPO 1XF objective (115×) and an Olympus SDF PLAPO 2XPFC objective (230× magnification). Blue-Tac (Bostik, Inc., Wauwatosa, Wisconsin, USA) and molding clay (Sculpey, Polyform Products Company, Elk Grove Village, Illinois, USA) was used to stabilise specimens during imaging and observation. Stacks of bright field images were taken manually on an Olympus CX41 microscope with a Canon EOS 70D camera attached. Images were subsequently aligned and stacked using Zerene Stacker Version 1.04 Build T201706041920. Figures were created in Adobe Photoshop elements Version 3.1.

To prepare specimens for male genitalia dissection, metasomata were removed from point-mounted specimens and cleared with 35% H_2_O_2_ (Alfa Aesar) for 24 hours, then moved to 5% acetic acid (Distilled White Vinegar, Great Value) for 24 hours and subsequently moved to glycerol for dissection and short-term storage. Dissections were performed with #5 forceps (Rubis 5A-SA, Bioquip) and #2 insect pins (BioQuip). Male genitalia were then mounted between 1.5 mm thick, 24×50 mm cover glasses and imaged using an Olympus FV10i confocal laser scanning microscope. Following the methods of Mikó and Deans 2013, auto-fluorescence was collected between 470 and 670 nm with three channels assigned contrasting pseudocolors (420–520 nm, blue; 490–520 nm, green; and 570–670 nm, red). The images were processed in ImageJ (Version 2.0.0-rc-54/1.51g, Build 26f53fffab, Schindelin et al. 2015) using FIJI (Schindelin et al. 2012).

For the descriptions of male and female specimens, morphological characters (following Burks et al. 2016) were scored based on observations of point-mounted and glycerine-stored specimens. Specimen data, OTU concepts, natural language phenotypes and images were compiled in the online database MX (http://purl.oclc.org/NET/mx-database) which was used to render the Diagnosis, Description, Material Examined and Etymology sections. Anatomical terms follow the Hymenoptera Anatomy Ontology (Yoder et al. 2010).

Semantic statements were generated in Protégé Version 5.0 beta-15 following the methods of Trietsch et al. (2015). Statements were written in OWL Manchester Syntax (see examples from Balhoff et al. 2014, Balhoff et al. 2013, Mikó et al. 2015, Mikó et al. 2014). All definitions and descriptions of morphological structures were mapped to classes in phenotype-focused ontologies, including: Hymenoptera Anatomy Ontology (HAO), Phenotypic Quality Ontology (PATO), Biospatial Ontology (BSPO), OBO Relation Ontology (RO), Ontology for Biomedical Investigations (OBI) and Information Artifact Ontology (IAO); these ontologies are available at http://www.ontobee.org (Xiang et al. 2011). By standardising taxonomic data through ontology-based semantic representation, we aim to facilitate future systematic work by facilitating the integration of taxonomic data sets from different sources, expediting computerised searches across these data (Balhoff et al. 2013, Deans et al. 2012, Mikó et al. 2014).

All figures, media files, protocols, semantic statements and supplementary files are available on figshare at https://figshare.com/projects/Unique_extrication_structure_in_a_new_megaspilid_Dendrocerus_scutellaris_Hymenoptera_Megaspilidae_Trietsch_and_Mik_/27007. The taxonomic treatment file generated from MX (Suppl. material 2) and the OWL file containing all semantic statement annotations (Suppl. material 3) are also available on GitHub at https://github.com/hymao/hymao-data/blob/master/scutellaris_semantic_statements.owl and https://github.com/hymao/hymao-data/blob/master/scutellaris_mx_taxonomic_treatment.owl.

## Taxon treatments

### Dendrocerus scutellaris

Trietsch & Mikó sp. n.

urn:lsid:zoobank.org:act:FAC23EE4-1B59-48EE-B20A-65472BCD9589

#### Materials

- **Type status:** Holotype. **Occurrence:** catalogNumber: NHMUK010812028; recordedBy: D Janzen & I. Gauld; individualCount: 1; sex: male; lifeStage: adult; occurrenceID: http://grbio.org/institution/frost-entomological-museum-penn-state-university/53e1ac2c-ea1c-4ff2-8649-5017127e66b9; **Taxon:** scientificName: Dendrocerus scutellaris; kingdom: Animalia; phylum: Arthropoda; class: Insecta; order: Hymenoptera; family: Megaspilidae; genus: Dendrocerus; specificEpithet: scutellaris; taxonRank: species; **Location:** country: Costa Rica; countryCode: CR; stateProvince: Guanacaste; verbatimLocality: COSTA RICA: Guanacaste Pr.: Santa Rosa N. P.: 300m Bosque San Emilio; verbatimElevation: 300m; **Identification:** identifiedBy: Carolyn Trietsch; dateIdentified: 2017; **Event:** eventDate: 1985-04-27/5-11; verbatimEventDate: 27.iv-11.v.1985; eventRemarks: sample SE.6.C. BMNH(E) 2008-87; **Record Level:** language: en; institutionID: http://biocol.org/urn:lsid:biocol.org:col:34665; institutionCode: NHMUK; collectionCode: Insects; basisOfRecord: PreservedSpecimen
- **Type status:** Paratype. **Occurrence:** catalogNumber: NHMUK010812044; recordedBy: D Janzen & I. Gauld; individualCount: 1; sex: female; lifeStage: adult; occurrenceID: http://grbio.org/institution/frost-entomological-museum-penn-state-university/19410856-81ec-4ea2-b003-861d28be9fab; **Taxon:** scientificName: Dendrocerus scutellaris; kingdom: Animalia; phylum: Arthropoda; class: Insecta; order: Hymenoptera; family: Megaspilidae; genus: Dendrocerus; specificEpithet: scutellaris; taxonRank: species; **Location:** country: Costa Rica; countryCode: CR; stateProvince: Guanacaste; verbatimLocality: COSTA RICA: Guanacaste Pr.: Santa Rosa N. P.: 300m Bosque San Emilio; verbatimElevation: 300m; **Identification:** identifiedBy: Carolyn Trietsch; dateIdentified: 2017; **Event:** eventDate: 1985-10-5/26; verbatimEventDate: 5-26.x.1985; eventRemarks: sample SE.6.C. BMNH(E) 2008-87; **Record Level:** language: en; institutionID: http://biocol.org/urn:lsid:biocol.org:col:34665; institutionCode: NHMUK; collectionCode: Insects; basisOfRecord: PreservedSpecimen
- **Type status:** Paratype. **Occurrence:** catalogNumber: NHMUK010812045; recordedBy: D Janzen & I. Gauld; individualCount: 1; sex: female; lifeStage: adult; occurrenceID: http://grbio.org/institution/frost-entomological-museum-penn-state-university/60cf3fa8-009b-4b7e-a529-fc933f737604; **Taxon:** scientificName: Dendrocerus scutellaris; kingdom: Animalia; phylum: Arthropoda; class: Insecta; order: Hymenoptera; family: Megaspilidae; genus: Dendrocerus; specificEpithet: scutellaris; taxonRank: species; **Location:** country: Costa Rica; countryCode: CR; stateProvince: Guanacaste; verbatimLocality: COSTA RICA: Guanacaste Pr.: Santa Rosa N. P.: 300m Bosque San Emilio; verbatimElevation: 300m; **Identification:** identifiedBy: Carolyn Trietsch; dateIdentified: 2017; **Event:** eventDate: 1985-07-5/8-3; verbatimEventDate: 13.vii-3.viii.1985; eventRemarks: sample SE.6.C. BMNH(E) 2008-87; **Record Level:** language: en; institutionID: http://grbio.org/cool/29fv-ztxs; institutionCode: PSUC; collectionCode: Insects; basisOfRecord: PreservedSpecimen
- **Type status:** Paratype. **Occurrence:** catalogNumber: NHMUK010812030; recordedBy: D Janzen & I. Gauld; individualCount: 1; sex: male; lifeStage: adult; occurrenceID: http://grbio.org/institution/frost-entomological-museum-penn-state-university/50d80e83-a282-43c7-9514-eb821ee2b64f; **Taxon:** scientificName: Dendrocerus scutellaris; kingdom: Animalia; phylum: Arthropoda; class: Insecta; order: Hymenoptera; family: Megaspilidae; genus: Dendrocerus; specificEpithet: scutellaris; taxonRank: species; **Location:** country: Costa Rica; countryCode: CR; stateProvince: Guanacaste; verbatimLocality: COSTA RICA: Guanacaste Pr.: Santa Rosa N. P.: 300m Bosque San Emilio; verbatimElevation: 300m; **Identification:** identifiedBy: Carolyn Trietsch; dateIdentified: 2017; **Event:** eventDate: 1985-07-5/8-3; verbatimEventDate: 13.vii-3.viii.1985; eventRemarks: sample SE.6.C. BMNH(E) 2008-87; **Record Level:** language: en; institutionID: http://biocol.org/urn:lsid:biocol.org:col:34665; institutionCode: NHMUK; collectionCode: Insects; basisOfRecord: PreservedSpecimen

#### Description

Body length universal: 2.6-2.7 mm.

**Colouration**: Colour hue pattern: head and mesosoma black; metasoma, mouthparts, legs and scape except for the basal part dark brown; base of scape light brown. Colour intensity pattern: proximal part of scape lighter than the rest of the scape.

**Head**: Cephalic size (csb): mean: 750-1100 μm. Head height (lateral view) vs. eye height (anterior view): HH:EHf=1.25-1.75. Head height vs. head length: HH:HL=1.2-1.5. Head width vs. interorbital space: HW:IOS=1.6-1.9. Head width vs. head height: HW:HH=1.5-2.0. Male ocular ocellar line vs. lateral ocellar line: OOL:LOL=2.1-2.6. Male ocular ocellar line vs. posterior ocellar line: OOL:POL=0.95-1.0. Female ocular ocellar line vs. lateral ocellar line: OOL 1.6–2.5 x as long as LOL. Anterior ocellar fovea shape: fovea not extended ventrally into facial sulcus. Occipital carina sculpture: crenulate. Median flange of occipital carina count: absent. Preoccipital carina count: present. Preoccipital lunula count: present. Preoccipital furrow count: present. Preoccipital furrow anterior end: preoccipital furrow ends inside ocellar triangle. Dorsal margin of occipital carina vs. dorsal margin of lateral ocellus in lateral view: occipital carina is ventral to lateral ocellus in lateral view. Transverse scutes on upper face count: absent. Rugose region on upper face count: present. Rugose sculpturing on head and mesosoma count: present. Facial pit count: facial pit present. Intertorular carina count: present. Ventral margin of antennal rim vs. dorsal margin of clypeus: not adjacent. Median region of intertorular area shape: concave. Subtorular carina count: present. Torulo-clypeal carina count: present. Supraclypeal depression count: present. Supraclypeal depression structure: absent medially, represented by two grooves laterally of facial pit. Antennal scrobe count: absent. Mandibular tooth count: 1. Mandibular lancea count: absent. Distal edge of mandible: flat.

**Antennae**: Male flagellomeres shape: branched. Male scape length vs combined length of F1+F2: longer or equal. 6th male flagellomere length vs. width, “sensillar” view: elongate, more than 2x as long as wide. Male flagellomere branches count: 7 branches ; 8 branches . Branch of male flagellomere 5 length compared to flagellomere 6: longer than length of flagellomere 6. Branch of male F5 length vs. length of male F5: longer than length of flagellomere 5. Male F6 length vs. combined length of F7+F8: shorter than length of flagellomere 7+8. Sensillar patch of the male flagellomere pattern: F7-F9. Basal resilin-rich area of male antennal branches count: absent. Female F1 length vs. pedicel length: 1.0-1.2. Female ninth flagellomere length: F9 less than F7+F8.

**Mesosoma and Metasoma**: Ventrolateral invagination of the pronotum count: present. Notaulus posterior end location: adjacent to transscutal articulation. Speculum ventral limit: not extending ventrally of pleural pit line. Mesoscutellar comb count: present. Mesoscutal length vs. anterior mesoscutal width: MscL/AscW=1.2–2.0. Anterior mesoscutal width vs. posterior mesoscutal width: AscW/PscW=0.7-0.9. Median mesoscutal sulcus posterior end: adjacent to transscutal articulation. Axillular carina count: absent. Scutoscutellar sulcus vs. transscutal articulation: adjacent. Mesometapleural sulcus count: present. Metapleural carina count: present. Anteromedian projection of the metanoto-propodeo-metapecto-mesopectal complex count: present. Anteromedian projection of the metanoto-propodeo-metapecto-mesopectal complex shape: Bifurcated.

**Male Genitalia**: Distal margin of male S9 shape: convex. Proximolateral corner of male S9 shape: blunt. Proximodorsal notch of cupula count: absent. Gonostyle/volsella complex proximodorsal margin shape: with deep concavity medially. Submedian conjunctiva on distoventral margin of gonostyle/volsella complex: length (range of fusion of parossiculus/parossiculus complex from gonostipes): more than 4/5. Apical parossiculal seta number: one. Dorsal apodeme of penisvalva count: absent. Distal projection of the penisvalva count: absent. Sensillar plate of the aedeagus shape: distinctly less than half as wide as the male genitalia. Distal projection of the parossiculus count: present. Dorsomedian conjunctiva of the gonostyle-volsella complex count: absent. Cupula length vs. gonostyle-volsella complex length: cupula less than 1/2 the length of gonostyle-volsella complex in lateral view. Parossiculus count (parossiculus and gonostipes fusion): present (not fused with the gonostipes). Distoventral submedian corner of the cupula count: absent. Harpe length: harpe shorter than gonostipes in lateral view.

#### Diagnosis

**Figure 1.**
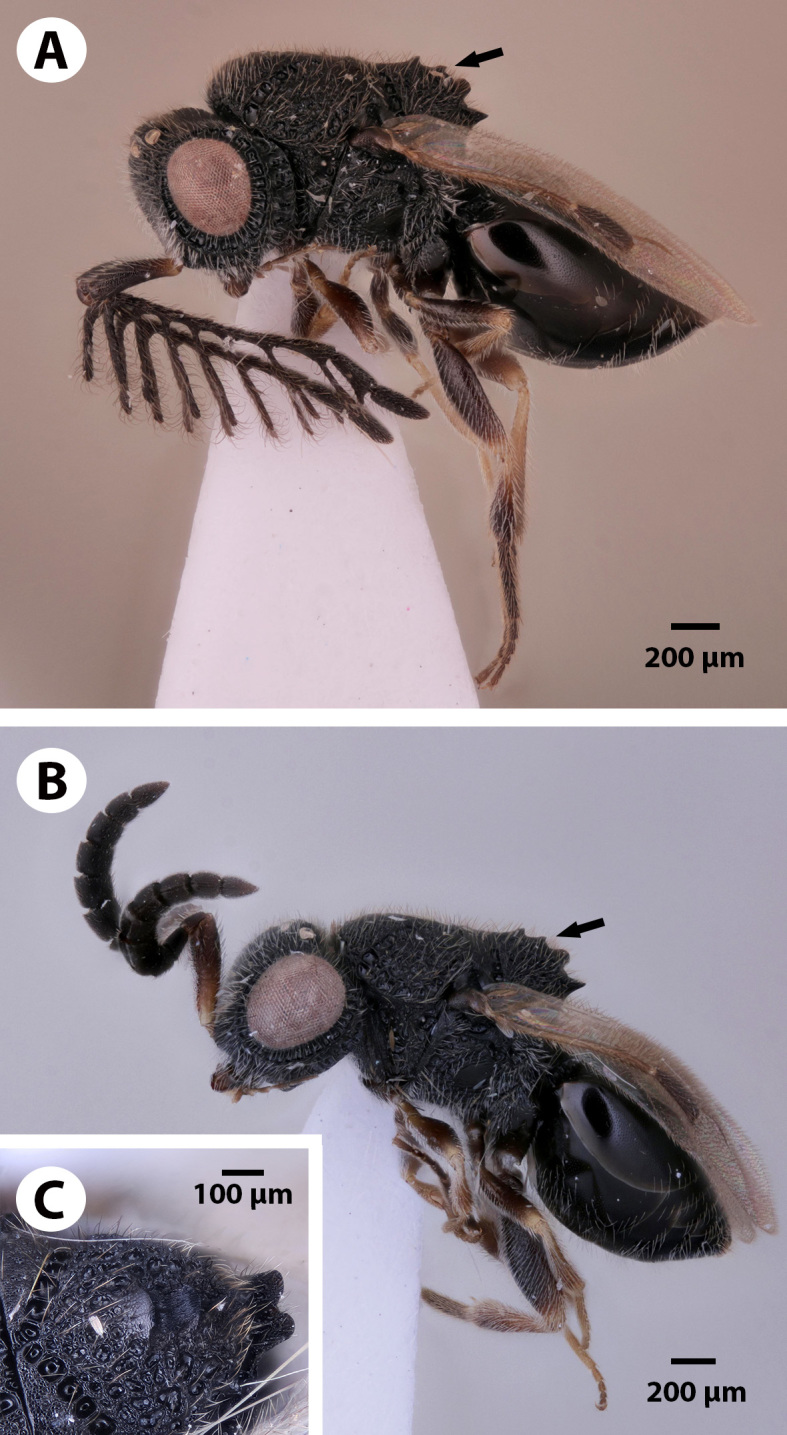
*Dendrocerus scutellaris* habitus, with arrows pointing to the mesoscutellar comb. A. Male holotype (NHMUK010812028). B. Female paratype (NHMUK010812044). C. Dorso-lateral view of the mesoscutellar comb (female paratype NHMUK010812045).

**Figure 2.**
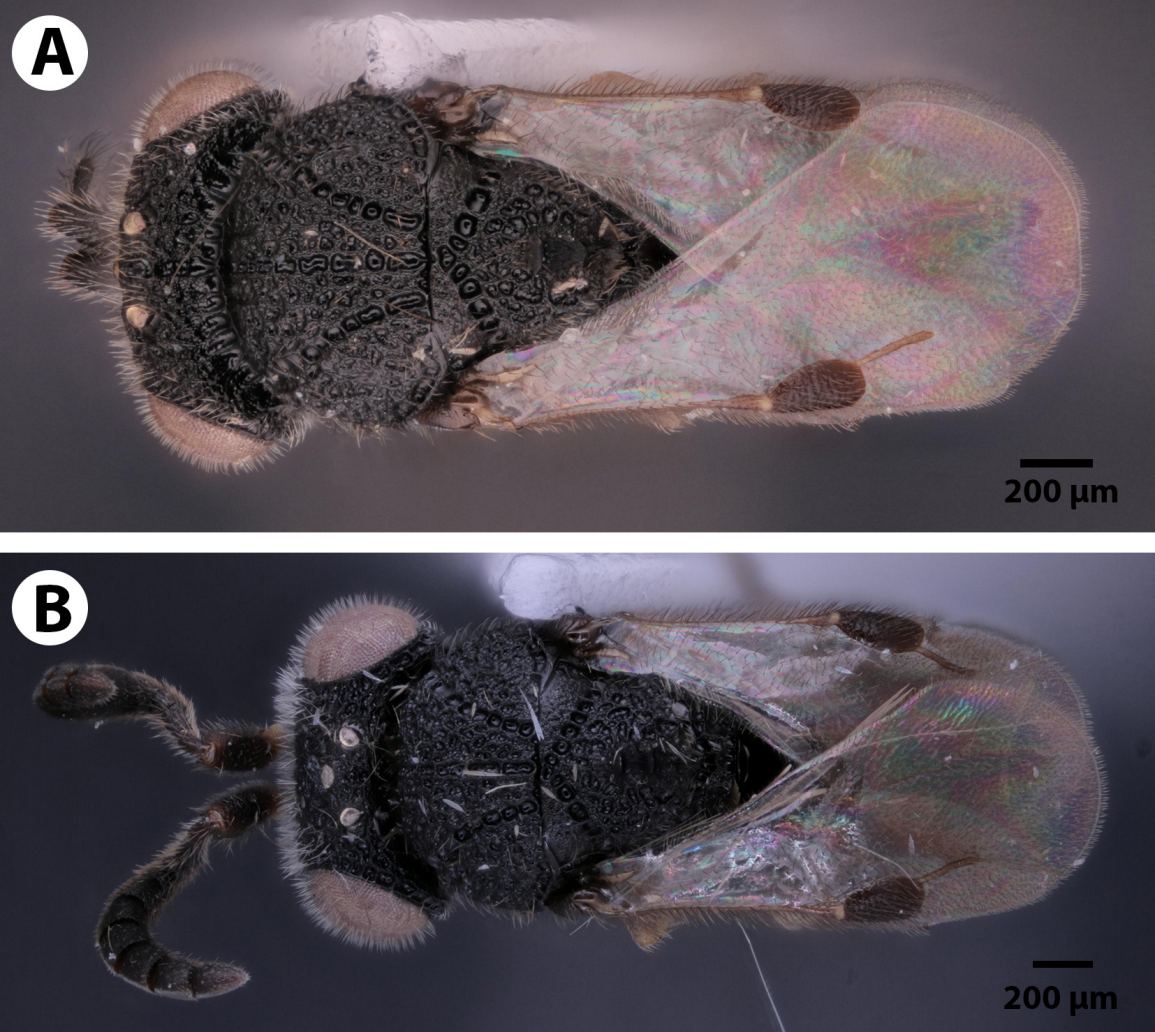
Dorsal view of *Dendrocerus scutellaris.* A. Male holotype (NHMUK010812028). B. Female paratype (NHMUK010812044).

**Figure 3.**
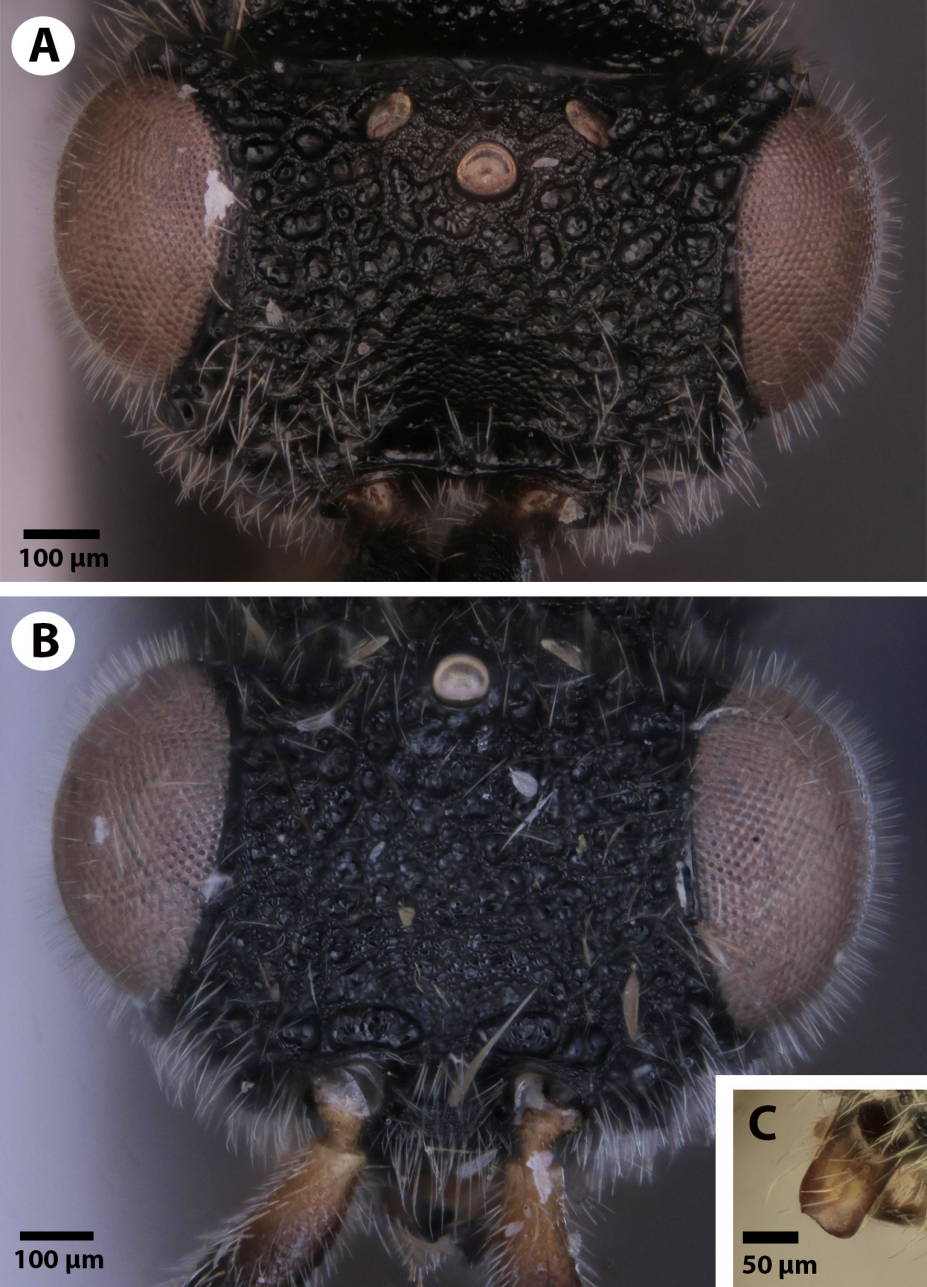
Frons of *Dendrocerus scutellaris.* A. Male holotype (NHMUK010812028). B. Female paratype (NHMUK010812044). C. Image showing the flat distal edge of the mandible from the male paratype (NHMUK010812030).

**Figure 4.**
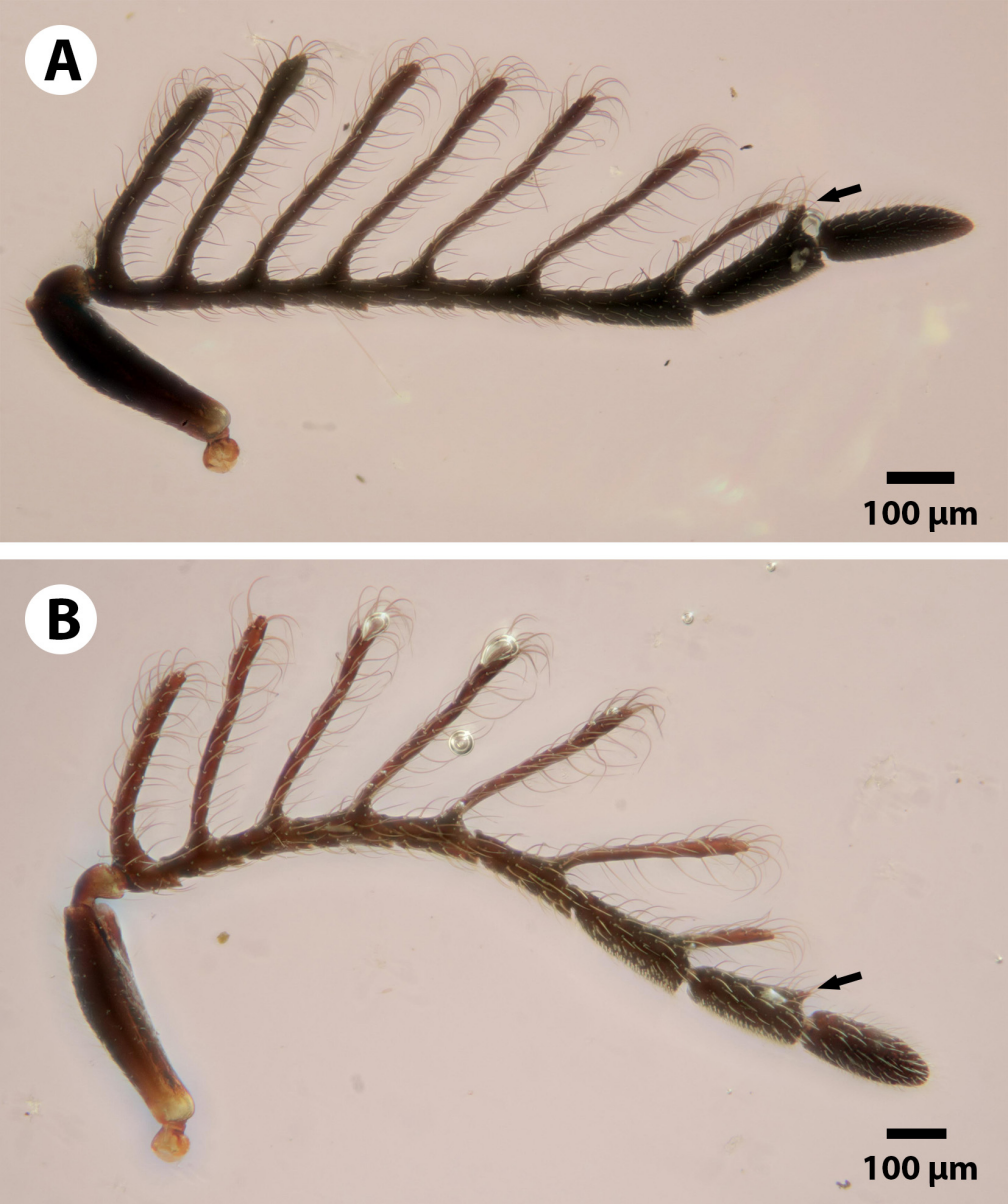
Antennae of the two known male specimens of *Dendrocerus scutellaris*, with arrows pointing to the variable eighth branch. The branch is more developed in the male holotype (A; NHMUK010812028) than in the male paratype (B; Male NHMUK010812030).

**Figure 5.**
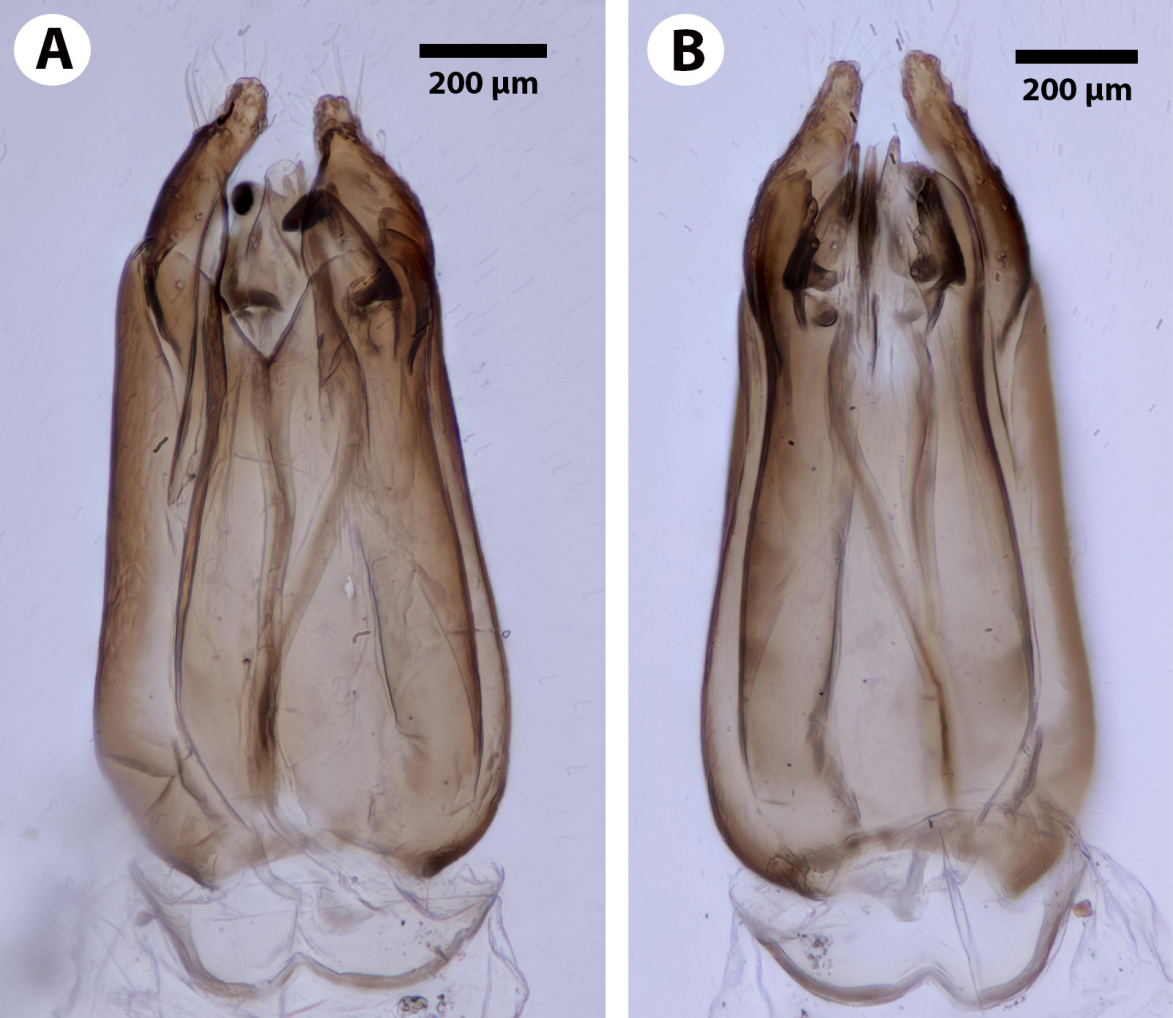
Genitalia of the male holotype (NHMUK010812028). A. Dorsal view. B. Ventral view.

**Figure 6.**
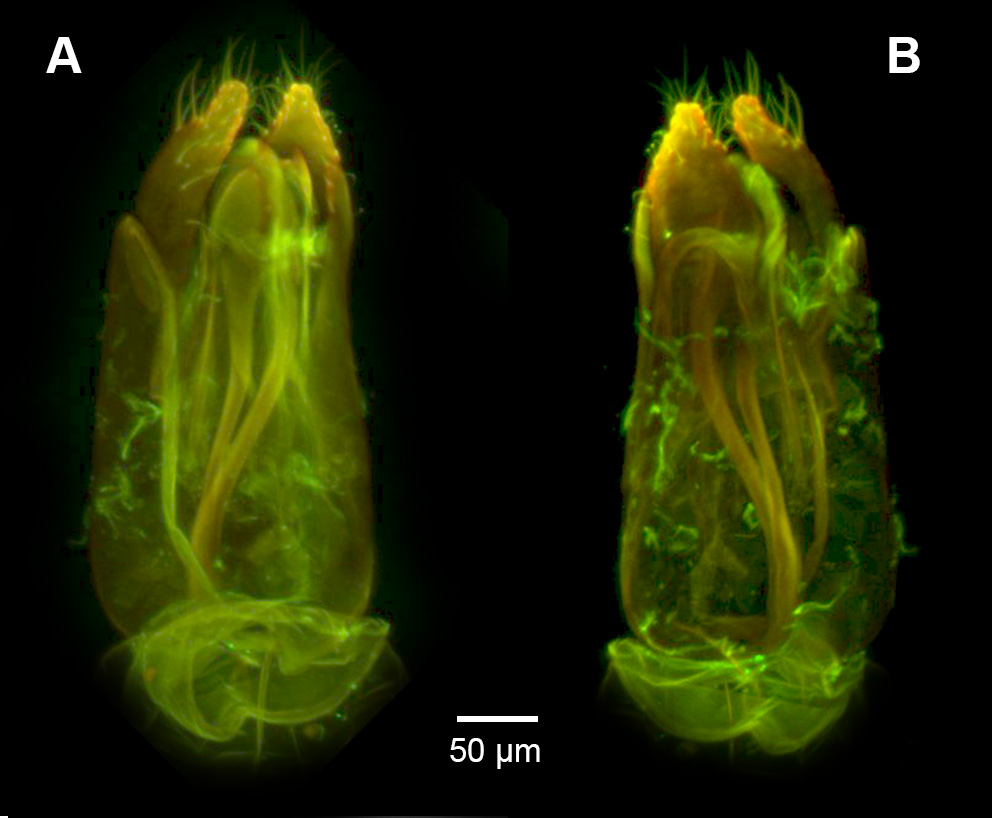
CLSM images of the genitalia of the male holotype (NHMUK010812028). A. Ventral view. B. Dorsal view.

*Dendrocerus scutellaris* (Figs 1, 2, 3, 4, 5, 6) belongs to the *Dendrocerus halidayi* species group (Dessart 1995, Dessart 1999), based on the branched male flagellomeres, bifid anteromedian projection of the metanoto-propodeo-metapectal complex and the presence of parossiculal projections with 3 parossiculal setae. This species is distinguished from all other ceraphronoid species by the presence of the mesoscutellar comb, an anatomical cluster that is composed of a row of spines medially on the mesoscutellar-axillar complex. This species is also unique amongst Ceraphronoidea in that the distal edge of mandible is flat and not pointed.

#### Etymology

This species is named for the presence of the mesoscutellar comb, which is unique to this species and is not found in any other known ceraphronoid species.

#### Distribution

This species is only known from Costa Rica.

#### Figs 1-6

## Discussion

*Dendrocerus scutellaris* belongs to the *halidayi* species-group, which is characterised by the presences of flabellate antennae in males (Dessart 1995, Dessart 1999). This new species is one of the few *Dendrocerus* species that also possesses a bifurcated anteromedian projection of the metanoto-propodeo-metapecto-mesopectal complex (Fig. 1). This character is also present in *D. africanus* and *D. australicus* Dodd 1914, both members of the *halidayi* species-group Dessart 1995, Dessart 1999. Based on the morphological features which these species share in common, it is hypothesised that this species is the closest known living relative to *Dendrocerus scutellaris*.

*D. scutellaris* is unique amongst members of the *halidayi* species-group in that, while other species have up to six fully formed branches on the flagellomeres (Dessart 1999, Dessart 1995), *Dendrocerus scutellaris* has 7 fully formed branches with a variable eighth branch that is more developed in the holotype male specimen than in the paratype male (Fig. 4). The length of the seventh branch also varies between specimens; in the holotype, the branch is as long as the branch on the seventh flagellomere is as long as the seventh flagellomere, while in the paratype specimen, it is shorter than the seventh flagellomere. Though the number of flagellomeres with branches and branch length has been used as a character to describe new species of *Dendrocerus* in the past (Pezzini et al. 2014), *Dendrocerus scutellaris* clearly exhibits intraspecific variation in the number and length of the flagellar branches between both male type specimens. Thus, branch length and presence of apical branches should not be used exclusively to describe *Dendrocerus* species.

*Dendrocerus scutellaris* is distinguished from all other ceraphronoid species by the presence of a straight mandibular surface (Fig. 3) and the presence of the mesoscutellar comb (Fig. 1), a ridge of backward-facing dorsal projections present in both male and female specimens. Though nothing is known about the life history or host identity of this species, other species such as *Dendrocerus carpenteri* Curtis 1829 are known to parasitise braconid parasitoids inside of aphid mummies (Mackauer and Chow 2015). It is hypothesised that the function of the mesoscutellar comb is to aid in emergence and extrication of the adult from its host. The function of the mesoscutellar comb is further suggested by the fact that the distal surface of the mandible is straight instead of pointed, and thus presumably cannot be used for piercing or tearing of the pupal case. All other known Ceraphronoidea possess a pointed mandibular edge and lack the mesoscutellar comb; the presence of a straight mandibular edge in the only ceraphronoid with a mesoscutellar comb is not likely to be a coincidence.

## Supplementary Material

Supplementary material 1 Specimen Locality Information Data type: occurences
Brief description: A table listing all of the specimens used in this study and their associated locality and repository information.
File: oo_170898.xlsx Carolyn Trietsch, István Mikó, David G. Notton, Andrew R. Deans

Supplementary material 2 MX Taxonomic Treatment File Data type: n3 File
Brief description: The taxonomic treatment file generated from MX used to write semantic statements.
File: oo_170907.n3 Carolyn Trietsch, István Mikó, David G. Notton, Andrew R. Deans

Supplementary material 3 Semantic Statement Phenotype Annotations Data type: OWL file
Brief description: The file containing all of the semantic statement phenotype annotations.
File: oo_170908.owl Carolyn Trietsch, István Mikó, David G. Notton, Andrew R. Deans

XML Treatment for Dendrocerus scutellaris
